# Prediction of Perforated and Nonperforated Acute Appendicitis Using Machine Learning-Based Explainable Artificial Intelligence

**DOI:** 10.3390/diagnostics13061173

**Published:** 2023-03-19

**Authors:** Sami Akbulut, Fatma Hilal Yagin, Ipek Balikci Cicek, Cemalettin Koc, Cemil Colak, Sezai Yilmaz

**Affiliations:** 1 Department of Surgery, Liver Transplant Institute, Inonu University Faculty of Medicine, 244280 Malatya, Turkey; 2 Department of Biostatistics, and Medical Informatics, Inonu University Faculty of Medicine, 44280 Malatya, Turkey

**Keywords:** nonperforated acute appendicitis, perforated acute appendicitis, predictive markers, machine learning, explainable artificial intelligence

## Abstract

Background: The primary aim of this study was to create a machine learning (ML) model that can predict perforated and nonperforated acute appendicitis (AAp) with high accuracy and to demonstrate the clinical interpretability of the model with explainable artificial intelligence (XAI). Method: A total of 1797 patients who underwent appendectomy with a preliminary diagnosis of AAp between May 2009 and March 2022 were included in the study. Considering the histopathological examination, the patients were divided into two groups as AAp (*n* = 1465) and non-AAp (NA; *n* = 332); the non-AAp group is also referred to as negative appendectomy. Subsequently, patients confirmed to have AAp were divided into two subgroups: nonperforated (*n* = 1161) and perforated AAp (*n* = 304). The missing values in the data set were assigned using the Random Forest method. The Boruta variable selection method was used to identify the most important variables associated with AAp and perforated AAp. The class imbalance problem in the data set was resolved by the SMOTE method. The CatBoost model was used to classify AAp and non-AAp patients and perforated and nonperforated AAp patients. The performance of the model in the holdout test set was evaluated with accuracy, F1- score, sensitivity, specificity, and area under the receiver operator curve (AUC). The SHAP method, which is one of the XAI methods, was used to interpret the model results. Results: The CatBoost model could distinguish AAp patients from non-AAp individuals with an accuracy of 88.2% (85.6–90.8%), while distinguishing perforated AAp patients from nonperforated AAp individuals with an accuracy of 92% (89.6–94.5%). According to the results of the SHAP method applied to the CatBoost model, it was observed that high total bilirubin, WBC, Netrophil, WLR, NLR, CRP, and WNR values, and low PNR, PDW, and MCV values increased the prediction of AAp biochemically. On the other hand, high CRP, Age, Total Bilirubin, PLT, RDW, WBC, MCV, WLR, NLR, and Neutrophil values, and low Lymphocyte, PDW, MPV, and PNR values were observed to increase the prediction of perforated AAp. Conclusion: For the first time in the literature, a new approach combining ML and XAI methods was tried to predict AAp and perforated AAp, and both clinical conditions were predicted with high accuracy. This new approach proved successful in showing how well which demographic and biochemical parameters could explain the current clinical situation in predicting AAp and perforated AAp.

## 1. Introduction

Acute appendicitis (AAp) is one of the most common causes of admission to emergency departments due to abdominal pain [1,2,3,4]. Obstruction of the lumen of the appendix vermiformis for any reason is the most important triggering factor for initiating the inflammatory process in AAp [1,2,3,4]. Although AAp can be seen at any age, it is mostly seen in the second decade of life, associated with lymphoid tissue development. In addition to studies showing that AAp is more common in men, there are studies showing that it is more common in women, but these rates vary between 1–1.3 times [5,6]. Although many studies have stated that the incidence of AAp varies between 100 and 151 per 100 thousand people, an epidemiological study using global health data showed that the actual incidence of AAp had increased to 228 per 100 thousand people [1,2,4,6]. The lifetime risk of having AAp is approximately 8.6% for men and 6.7% for women [1,2,4,7,8]. On the other hand, epidemiological studies show that men and women have a 12% and 23% risk of having an appendectomy at any time in their lives, respectively [1,4].

Although it has been claimed in recent studies that conservative treatment consisting of a combination of antibiotics and close follow-up is advantageous in patients with AAp, open or laparoscopic appendectomy continues to be the gold standard treatment in the treatment of AAp [9,10]. Therefore, performing an appendectomy as soon as possible after the diagnosis of AAp is confirmed to minimize the risk of perforation due to delays. This decision is made by evaluating the patient’s history, examination findings, and biochemical and radiological diagnostic methods.

Negative appendectomy (NA; non-AAp) and perforated Aap are the most emphasized issues in studies on Aap. As is known, only 50–67% of patients with Aap have typical Aap signs and symptoms [11,12,13]. Therefore, additional tests are needed to confirm the diagnosis in at least one-third of the patients [11,12,13]. Globally, it has been shown that the rates of non-Aap vary between 15% and 50%, but in recent years, the rates of non-Aap have decreased to below 10% with the more frequent use of laparoscopy and advanced radiological instruments [1,7,12,13]. Similarly, while epidemiological studies have shown that the incidence of perforated AAp varies between 19.4 and 27.2 per 100 thousand people [1,5], center-based studies have shown that perforated AAp rates vary between 16% and 46%. In current studies, it is seen that the rates of perforated AAp fall below 10%, just like non-AAp [1,7].

As mentioned above, diagnosis delay can cause complications related to AAp (perforation, abscess, free peritonitis, plastrone, pylephlebitis) and cause severe morbidity and mortality, especially in patient groups with comorbidities [14]. Delay in diagnosis not only causes complications in patients, it can also cause long-term hospitalization, job loss, cost increase due to additional tests, and psychosocial problems [15].

Perforated AAp and non-AAp are like the two ends of the seesaw, so when you act sensitively to lower one, the ratio of the other increases. In other words, finding the balance and reducing both to optimal levels can only be possible with evidence-based studies and predictions. Scientifically, scoring systems consisting of anamnesis, physical examination findings, biochemical blood parameters and radiological instruments and their combination (Alvarado score, Adult appendicitis score (AAS), Pediatric appendicitis score (PAS), Appendicitis inflammatory response (AIR) score, RIPASA score) are frequently used methods to solve the two problems we mentioned [16,17].

Radiological examinations and biochemical blood parameters, direct or indirect indicators of inflammation, are the most commonly used methods for predicting AAp-related complications and avoiding non-AAp. Ultrasonography (US) and computed tomography (CT) are widely used as radiological examinations [2,11]. However, the most important disadvantages of radiological methods are that they are operator dependent and not always accessible in emergency conditions [11]. Although many academic studies have examined cytokines (IL-6, IL-1β, TNF-α, IL-10, IP-10, MIP-1α) in the blood and serotonin metabolites (5-HIAA) in the urine, these tests are rarely routinely used in the diagnosis of AAp [18,19], because these examinations are both not cost-effective and have very controversial requirements. However, the complete blood count parameters (leukocyte, neutrophil, lymphocytes, platelets, platelet derivatives), which are among the routine biochemical blood parameters, as well as markers such as total bilirubin (TBil), C-Reactive protein (CRP) and procalcitonin, vary depending on the existence and severity of inflammation, and their clinical use is efficient [1,2,3,7,11].

All the reasons above show how important biochemical parameters are for predicting AAp and perforated AAp. The studies conducted to date have been performed using standard biostatistical analysis methods, in which clinical, radiological, and biochemical parameters can be used to predict AAp and perforated AAp. However, the results’ usability differs from center to center, and therefore the generalizability of the results to the population has been the topic of serious debate. All these factors have paved the way for the use of artificial intelligence (AI) models that will minimize the effect of the human factor in predicting AAp and perforated AAp.

The machine learning (ML) method, one of the AI methods that can be used in estimating AAp, has been demonstrated recently [11]. Unlike traditional statistical techniques, ML is a sub-field of AI that aims to make predictions about new observations by learning based on existing data. However, a significant problem in many state-of-the-art ML models is the lack of transparency, interpretability, and explainability. To overcome these shortcomings, explainable artificial intelligence (XAI) has recently started to attract more attention in clinical research. In this context, XAI deals with methods that aim to make ML models more understandable/interpretable by clinicians [20]. The Shapley Additive Explanations (SHAP) method, which is one of the XAI methods, determines the numerical values that show the direction and magnitude of the variable contributions to the estimations of the ML models and provides the visualization of the variable contributions [21]. This study aims to predict AAp and perforated AAp with ML models using patients’ clinical and biochemical blood parameters and interpret the results with SHAP, which is an XAI approach. From this point of view, it is thought that this study represents an important step forward for the use of XAI models for AAp, which is one of the most common reasons for admission to emergency services.

The main findings and contributions of this article are listed below:

- An ML model was created to accurately predict patients with AAp and perforated AAp.
- CatBoost performed well in distinguishing patients.
- With the SHAP method, it was determined that high total bilirubin, WBC, Neutrophil, WLR, NLR, CRP and WNR values and low PNR, PDW and MCV values increased the prediction of AAp biochemically.
- It was observed that high CRP, Age, Total Bilirubin, PLT, RDW, WBC, MCV, WLR, NLR and Neutrophil values and low Lymphocyte, PDW, MPV and PNR values increased the prediction of perforated AAp.
- The importance of the SHAP-based methodology was examined to explain the model, which can assist clinicians in diagnosing AAp and perforated AAp.
- ML and SHAP are useful in diagnosing and treating AAp and perforated AAp, future treatment goals, and personalized medication administration.

## 2. Materials and Methods

### 2.1. Study Design and the Related Dataset

Between May 2009 and March 2022, 1797 patients who underwent appendectomy with a preliminary diagnosis of AAp by the Department of Surgery of Inonu University Faculty of Medicine were divided into two main groups: AAp (*n* = 1465; 81.5%) and non-AAp (*n* = 332; 18.5%) based on the histopathological findings. Then, 1465 patients confirmed to AAp were then divided into two subgroups: nonperforated (*n* = 1161; 79.2%) and perforated AAp (*n* = 304; 20.8%). The presence of inflammatory cell infiltration in the appendectomy specimen without perforation was referred to as nonperforated AAp, perforation with inflammatory cell infiltration was referred to as perforated AAp, and the absence of inflammatory cell infiltration was referred to as non-AAp.

Routine biochemical parameters that are frequently used to predict AAp and its complications are as follows: white blood cell count (WBC), white blood cell–lymphocyte ratio (WLR), white blood cell–neutrophil ratio (WNR), neutrophil–lymphocyte ratio (NLR), C-reactive protein (CRP), platelet count (PLT), platelet–neutrophil ratio (PNR), platelet–lymphocyte ratio (PLR), platelet distribution width (PDW), mean platelet volume (MPV), total bilirubin (TBil), red blood cell distribution width (RDW), mean corpuscular hemoglobin (MCH) and mean corpuscular volume (MCV)

### 2.2. Data Preprocessing and Modeling

**Figure 1:**
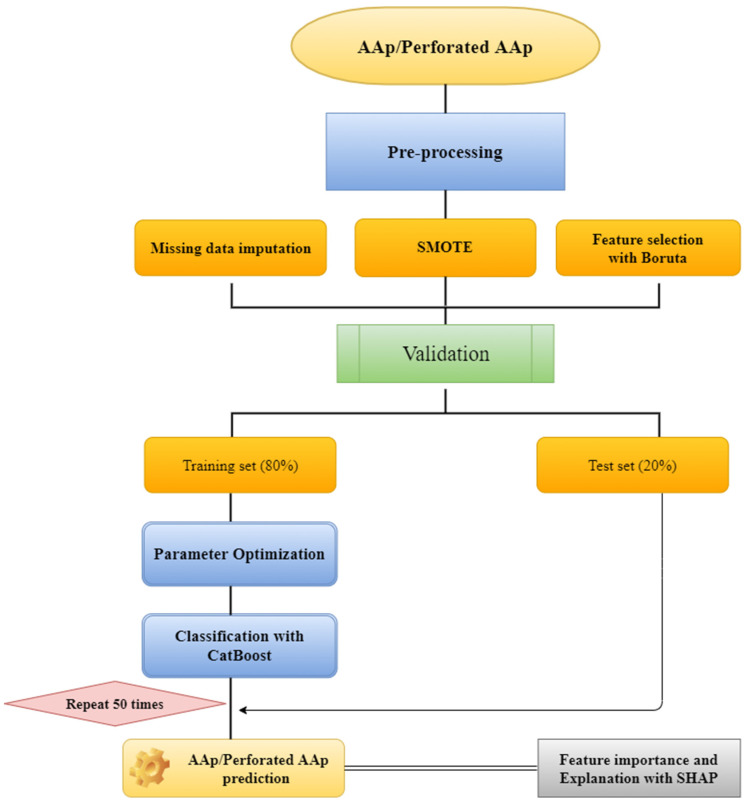
Diagram of the proposed method combining explainability and classifier.

The random forest method assigned the missing values in the data set. The Boruta feature selection method was used to determine the most essential variable (predicting factors) for AAp and subgroup (perforated AAp) prediction. The class imbalance problem in the data set used in the study was resolved with the SMOTE method. The data were split 80:20 into training and test sets. To obtain a more robust prediction model, avoid biased results and limit the problem of overfitting, the persistence method was repeated 50 times with different random seeds, and the average performance was calculated across these 50 times (Figure 1). The CatBoost model was used to predict patients with AAp and perforated AAp. The CatBoost model’s hyperparameters, which are important parameters that affect the performance of the prediction models, were optimized using the grid search method and 10-fold cross validation with 5 replicates. The model’s performance was evaluated with respect to accuracy, F1-score, sensitivity, specificity, and area under the receiver operator curve (AUC). The SHAP method, one of the XAI approaches, was used to interpret the model results. The methods used in the study are explained in the subtitles. Figure 1 provides an overview of the methodology.

### 2.3. Random Forest Missing Value Imputation

RF calculates a (nxn) proximity matrix to evaluate the similarity of observations in missing value imputation. The matrix’s off-diagonal elements show how two different observations are comparable. RF performs an iterative procedure for imputation based on these proximity values by performing the following steps: After employing median imputation, an initial forest is created, and proximities are then computed. A proximity-based weighted mean is used to determine new imputed values. A new forest is constructed using this updated data set, yielding new proximities and imputed values [22].

### 2.4. Synthetic Minority Over-Sampling Technique (SMOTE)

SMOTE is one of the oversampling approaches suggested by Chawla et al. [23]. Based on feature space similarities between existing minority observations, and the SMOTE algorithm creates synthetic data. SMOTE randomly chooses a minority class observation (a) and locates its k-nearest minority class neighbors in order to develop new synthetic minority class observations. Then, one of the k-nearest neighbor elements (b) is randomly selected, and the synthetic observation is derived by constructing a line segment connecting a to b in the feature space. A convex combination of two chosen observations a and b yields synthetic observations [24].

### 2.5. Boruta Feature Selection

The Boruta algorithm is a feature selection algorithm that is placed under the RF classification method. Boruta employs shadow features, which are copies of the original features. The shadow features are randomly assigned to objects; therefore, decision trees are generated based on the shadow features. In addition, this algorithm considers multi-variable relationships and can investigate interactions between variables [25].

### 2.6. CatBoost

CatBoost is a new gradient boosting technique presented by Prokhorenkova et al. [26] and Dorogush et al. [27] that works with categorical features with the least information loss [28]. To begin, it employs ordered boosting, a highly efficient variation of gradient boosting methods, to address the issue of target leaking. Second, this approach works well with tiny datasets. Third, CatBoost is capable of handling categorical features. This processing is often conducted during the preprocessing phase and consists primarily of substituting the original categorical variables with one or more numerical values. Furthermore, Bakhareva et al. [29] discovered that CatBoost might be successfully applied to various data kinds and formats. Another feature of the approach, as mentioned by Dorogush et al. [27], is that it uses random permutations to estimate leaf values while selecting the tree structure, hence avoiding the overfitting produced by typical gradient boosting algorithms.

### 2.7. Explainable Artificial Intelligence (XAI)

Computational learning theory and the study of pattern recognition led to the development of ML, a sub-branch of AI. ML is a collection of techniques and algorithms that can predict future events or classify data by learning patterns from previously collected data. Today, due to the complexity and large volume of data, human beings’ capacity to interpret them quickly is many times higher. From this point on, ML comes into play, enabling accurate forward-looking analysis of complex data [30]. In various industries, including the medical sciences, ML approaches have had significant success with predictive models in analyzing structured datasets. Most models developed by data scientists focus on the model’s accuracy in predicting the disease of interest, but models rarely explain these predictions. This is the black box feature of ML [31]. Traditional ML metrics such as AUC, accuracy, and recall may not be sufficient in many applications where the user must rely on ML system predictions. Understanding, explaining, and interpreting ML approaches is essential. While ML techniques have been in use for decades, their spread to areas such as healthcare has led to the greater emphasis on explanations in ML. The interpretability of model predictions is a priority for clinical practitioners regarding application and use. ML models that can explain why certain predictions are produced are called explainable AI models [32].

There are two types of XAI model: global interpretability and local interpretability. Global interpretability is the ability to examine the structure and parameters of a complex model and understand how the model works globally. On the other hand, local interpretability examines an individual prediction of a model locally and attempts to understand why the model made the decision it made. In this study, SHAP, one of the globally interpretable models, was used.

### 2.8. Shapley Additive Explanations (SHAP)

Difficulties in interpreting ML models and their predictions limit ML’s practical applicability and confidence. Model interpretability often depends on estimating the contribution of individual characteristics (independent variables) to the model’s results. Explainable approaches are needed to assist in the interpretation of ML models. To this end, the SHAP methodology was recently introduced [33].

SHAP is a method used in ML to explain the individual and global predictions of the model. The technique is theoretically based on optimal Shapley values. The technical definition of the Shapley value is the average marginal contribution of the value of a variable over all possible coalitions. In other words, Shapley values consider all potential estimates for an observation (sample) using all possible combinations of variables. Therefore, SHAP is a unified approach that provides global and local consistency and interpretability. In this context, it can be stated that the purpose of SHAP is to explain the estimation of any observation by calculating the contribution of each variable to the estimation [34]. The flow chart of all the methods used in the study is given in Figure 1.

### 2.9. Study Protocol and Ethics Committee Approval

This retrospective case–control study involving human participants was performed following the ethical standards of the institutional and national research committee and in accordance with the 1964 Helsinki Declaration and its later amendments or comparable ethical standards. First, the required permissions were obtained from the Directorate of Surgery. Then, ethical approval was obtained from the Inonu University Institutional Review Board (IRB) for non-interventional studies (2022/3481). STROBE (strengthening the reporting of observational studies in epidemiology) guidelines were utilized to assess the likelihood of bias and overall quality for this study [35].

## 3. Results

### 3.1. Acute Appendicitis versus Negative Acute Appendicitis

The total of 1797 patients in this retrospective study comprised 1465 (81.5%) patients with AAp and 332 (18.5%) patients with non-AAp. Of the patients, 993 (55.3%) were male (median age: 33 years; IQR: 23) and 804 (44.7%) were female (median age: 34 years; IQR: 26). The median age of patients with AAp was 33.1 years (IQR: 25), and the median age of patients with non-AAp was 33 years (IQR: 24).

Table 1 presents the accuracy, F1-score, sensitivity, specificity, and AUC values of the CatBoost model created for patients with AAp and non-AAp. When the performance criteria of the CatBoost model were examined for patients with AAp and non-AAp; accuracy 88.2% (85.6–90.8%), F1-score 88.7% (86.2–91.3%), sensitivity 84.2% (79.8–88%), specificity 93.2% (89.4–95.9%) and AUC 94.7% (91.3–96.2%) values were obtained.

**Figure 2:**
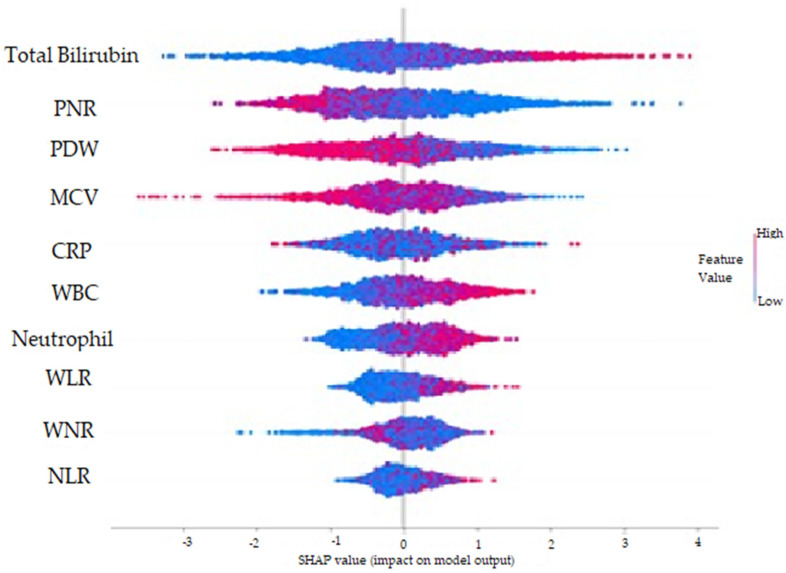
Feature importance plot according to normalized SHAP values for AAp.

**Figure 3:**
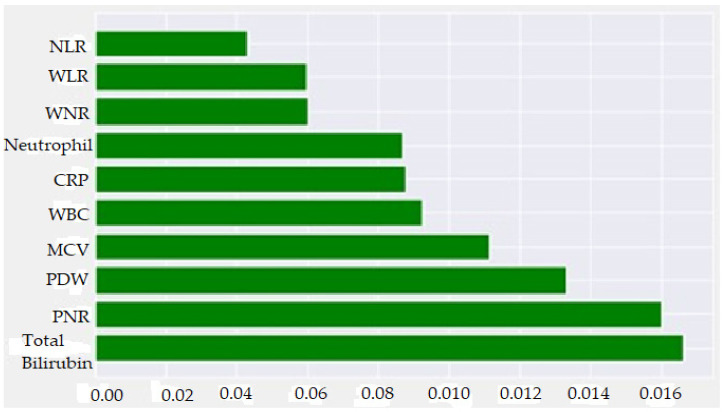
Graphical representation of the findings for the SHAP method for AAp.

The SHAP method was used to visually explain how the variables in the model affect the biochemical markers for AAp. Figure 2 shows possible markers evaluated by the normalized SHAP value and the importance levels of these markers for AAp. The aforementioned analysis findings showed that TBil, PNR, and PDW were the three most important predicting markers for AAp. Figure 3 was created by considering positive and negative SHAP values. A positive SHAP value indicates that the contribution to the target variable (AAp) is positive, and a negative SHAP value suggests that the contribution is negative. In addition, the variable’s value decreases as the points on the graph get closer to blue and increases as they get closer to pink. Therefore, higher TBil, WBC, Neutrophil, WLR, NLR, CRP, and WNR values and lower PNR, PDW, and MCV values indicate an increased risk of AAp. When the normalized SHAP values in Table 2 were examined, the five most predictive factors for AAp were TBil, PNR, PDW, MCV, and WBC. The explanatory powers of these five biochemical markers for AAp were 16.6%, 16%, 13.3%, 11.1%, and 9.2%, respectively.

### 3.2. Nonperforated AAp versus Perforated AAp

In this section, the 1465 patients with AAp were divided into two sub-groups according to their perforation status: perforated (n: 304; 20.8%) and nonperforated AAp (n: 1161; 79.2%). Of the patients, 847 (57.8%) were male (median age: 33 years; IQR: 22), and 618 (42.2%) were female (median age: 34 years; IQR: 26). The median age of patients with perforated AAp was 43 years (IQR: 32.75), and the median age of patients with nonperforated AAp was 32 years (IQR: 22).

Table 3 shows the accuracy, F1-score, sensitivity, specificity, and AUC values of the CatBoost model created for patients with perforated and nonperforated AAp. When the performance criteria of the developed CatBoost model were examined, the following values were obtained: accuracy 0.92 (89.6–94.5%), F1-score 91.1% (88.5–99.37%), sensitivity 94.1% (89.9–96.9%), specificity 90.5% (86.3–93.8%), and AUC 96.9% (90.4–98.7%).

**Figure 4:**
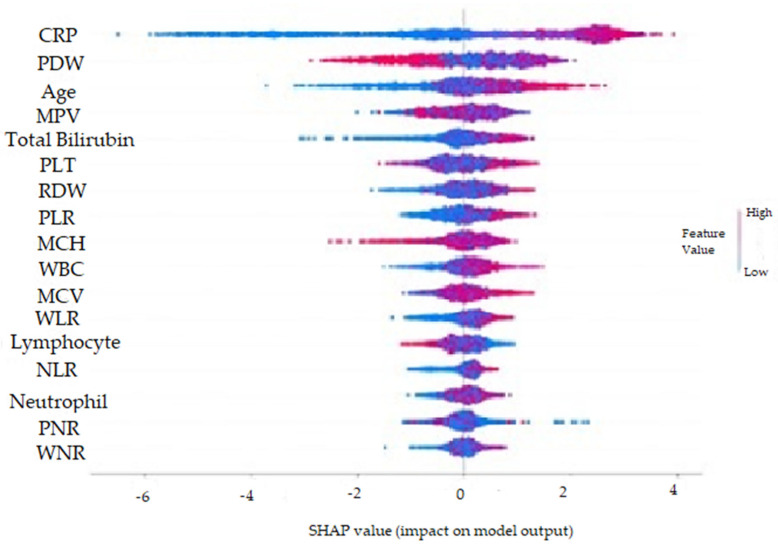
Feature importance plot according to normalized SHAP values for perforated AAp.

**Figure 5:**
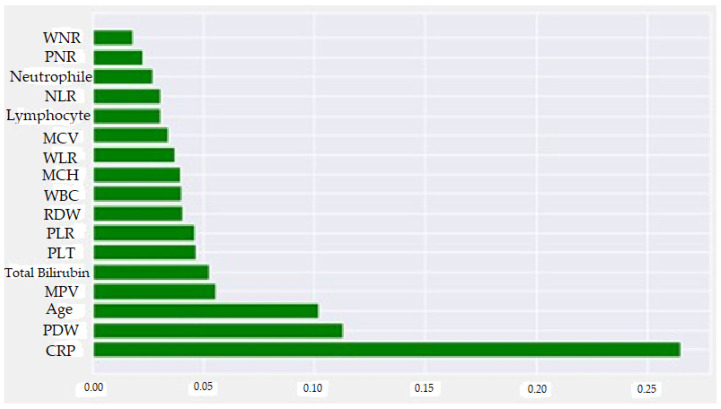
Graphical representation of the findings for the SHAP method for perforated AAp.

The SHAP method was used to visually explain how the variables in the model affect the biochemical markers for perforated AAp. Figure 4 shows the demographic and biochemical markers evaluated by the normalized SHAP value and the order of importance of these factors. These variable importances are given in ascending order. It can be said that the three most determinative factors for perforated AAp are CRP, PDW, and Age. Figure 5 was created by considering positive and negative SHAP values. Therefore, higher CRP, Age, TBil, PLT, RDW, WBC, MCV, WLR, NLR and Neutrophil values, and medium and low Lymphocyte, PDW, MPV, and PNR values indicate an increased risk of perforated AAp. As a result, it can be said that CRP value higher than 12.80 was the most critical determining biochemical marker for predicting perforated AAp. When the normalized SHAP values in Table 4 are examined, the five most determinative factors for perforated AAp were CRP, PDW, Age, MPV, and TBil. The explanatory powers of these biochemical markers for AAp were 26.5%, 11.3%, 10.2%, 5.5%, and 5.2%, respectively.

## 4. Discussion

Accurate classification and estimation of patients admitted to emergency services with a preliminary diagnosis of AAp using appropriate diagnostic algorithms prevents patients from being exposed to both unnecessary surgeries due to misdiagnosis and possible complications (perforation, abscess, etc.) may develop as a result of ignoring the actual patients. Furthermore, correct estimation minimizes the patient’s treatment cost and workforce loss.

ML is a subset of AI that uses statistical approaches to provide computer systems the capacity to learn and improve over time. ML, in particular, refers to AI tools that may update their models to improve predictions, resulting in a gradual performance improvement at the defined job. In theory, ML approaches may be used on any size dataset; nonetheless, more data gives more experience with which to train the model. In accordance with the ML working principle, these features are fed into computer models that can provide insights into the data, such as grouping similar observations into groups or forecasting certain events [36]. ML has attracted increasing medical research attention in recent years, with a wide range of applications being researched. Many studies have been performed analyzing different parts of the healthcare system, reporting improvements in ML engagement in illness prevention, screening, treatment, and prognosis prediction [37].

In the last decade, with the availability of large datasets and greater computing power, ML methods have achieved high performance in various situations. However, the main problem with many of the models used is the lack of transparency, explainability, and interpretability. In light of these problems, XAI has recently started attracting more attention. Briefly, XAI is the collection of methods or techniques that aim to make AI applications understandable by users. The aim of XAI is to make the computational inferences behind the decisions of AI, which has a process that is difficult to grasp in general, understandable by available users and researchers. Because ML often does not provide direct explanations for why or how predictions and results are obtained, it is difficult to show why model makes certain decisions. For this reason, explicable AI methods have been developed and applied to different models [20].

In this study, we aimed to predict AAp and its complications by combining ML and XAI models, which have been used in many areas of health care. In other words, from an epidemiological point of view, we aimed to minimize Type I (false positive) and Type II (false negative) error rates by using ML and XAI models.

To summarize the study presented here: firstly, the AAp and perforated AAp statuses of the patients were determined with the CatBoost model based on decision trees, which is one of the complex models, to increase prediction accuracy. Second, the global annotation method SHAP was used to avoid ambiguity of the complex CatBoost model. The CatBoost model could distinguish AAp patients from NA with an accuracy of 88.2% (85.6–90.8%) while discriminating perforated AAp patients from nonperforated AAp patients with an accuracy of 92% (89.6–94.5%). The main reason for the higher distinguishing accuracy in perforated AAp patients is the higher elevation of inflammation-related biochemical blood parameters during perforation compared to normal AAp.

In addition, through the proposed XAI approach, it was possible to list the most important biochemical blood parameters that can be used to predict AAp and perforated AAp. According to this evaluation, the most important biochemical blood parameters for AAp prediction were TBil, PNR, PDW, MCV, WBC, CRP, Neutrophil, WNR, WLR and NLR, respectively. The results of SHAP, which is the XAI approach, showed that the most important biochemical blood parameters detected could be used to predict high or low levels of AAp compared to normal. Accordingly, higher TBil, WBC, Neutrophil, WLR, NLR, CRP, and WNR values and lower PNR, PDW, and MCV values were associated with AAp. Similarly, the most important biochemical blood parameters for perforated AAp estimation were found to be CRP, PDW, age, MPV, TBil, PLT, PLR, RDW, WBC, MCH, WLR, MCV, Lymphocyte, NLR, Neutrophil, PNR, and WNR, respectively. SHAP results for perforated AAp revealed higher CRP, Age, TBil, PLT, RDW, WBC, MCV, WLR, NLR and Neutrophil values, and moderate and low Lymphocyte, PDW, MPV and PNR values were associated with perforated AAp. For AAp and perforated AAp, this can help physicians gain insight into the predictions made with the proposed CatBoost model to make a more accurate clinical diagnosis.

Some studies on the prediction of AAp by AI methods have been published in the literature. In one study, the support vector machine method was used to differentiate complicated AAp from non-complicated AAp, and the accuracy, sensitivity, specificity, and Matthews correlation coefficients were 83.56%, 81.71%, 85.33% and 67.32%, respectively [38]. In another study, Logistic Regression, Naive Bayes, Generalized Linear, Decision Tree, Support Vector Machine, Gradient Augmented Tree and Random Forest methods were used to predict whether appendicitis is acute or subacute. Among the methods, the random forest method gave the best results, with 83.75% accuracy, 84.11% precision, 81.08% sensitivity, and 81.01% specificity [39]. Akmese et al. [11] stated that the prediction success of various ML algorithms for the early diagnosis of AAp was compared, and the gradient boosted tree algorithm achieved the best success. This model achieved the best success, with an accuracy of 95.31%. In a study conducted with children and adolescents between the ages of 0 and 17 at a hospital in Germany, the complete blood counts of 590 patients with 473 appendicitis and 117 with negative histopathological findings were analyzed. In the study, AAp patients were estimated using ML methods. The model’s training was performed using the data of 35% of the patients, and 65% of the data were used for validation. In the study, 90% accuracy (with 93% sensitivity and 67% specificity) was obtained for the diagnosis of AAp [40]. Compared to both studies mentioned above, the accuracy of the current research in predicting AAp appears to be relatively lower (88.2% (85.6–90.8%)). This is because the biochemical parameters associated with AAp tend to increase more than normal due to the nature of the pediatric patients included in said studies. It is also important to evaluate the AUC along with accuracy. In our study, our model differentiated AAp patients from non-AAp patients with a very good AUC value of 94.7% (91.3–96.2%).

Most studies in the literature have used complex ML models for AAp prediction, but to the best of our knowledge, there are no studies on using XAI in predicting AAp and its complications. The primary contribution of the present study to the literature is its combination of ML and XAI. In addition, although most studies in the literature have examined AAp, there is limited research on perforated AAp. The secondary contribution of the present study to the literature is the interpretable estimation of perforated AAp using XAI.

Most studies conducted with conventional statistical methods reveal which parameters predict AAp and perforated AAp and show the relationship of changes in these parameters (such as fall and rise) with AAp. That is, conventional analyses fall short of demonstrating the significance of demographic and biochemical parameters and their ability to explain the clinical situation. On the other hand, models such as ML/XAI reveal the results of conventional statistical methods and the extent (%) of the parameters found to be significant to explain the clinical situation at hand.

Another study reporting on the current state of the art in postoperative risk estimation tackled the limitations of previous techniques and how they were used in practical settings. Additionally, the possibility of systematically incorporating machine learning models into health care in a broader sense and the future prospects beyond passive risk prediction were discussed [41]. Similarly, the current study investigated the prediction of perforated and nonperforated acute appendicitis using machine learning-based XAI, and evaluated potential implementations of the proposed algorithm integrated with XAI methods. Additionally, XAI techniques incorporated into AI/ML algorithms were of great importance for interpretable outcomes of the response variable associated with the explanatory factors. More explainable estimates could be obtained if different factors related to the disease and other AI/ML methods are used. This may limit the outputs of this study achieved from these models. The proposed approach with novel XAI methods may better highlight the results achieved from AI/ML methods.

### Limitations

As in other retrospective studies, this study has some limitations. First of all, most clinical data were excluded from the study, since most of the clinical characteristics of the patients (location of pain, duration, nausea, vomiting, anorexia) were not recorded in the hospital’s data processing system. Secondly, radiological data (US or CT) of approximately 11% of the patients included in this study could not be accessed. Excluding these patients whose radiological examinations could not be reached would decrease the sample size required for ML models and increase the class imbalance problem in the data set. For this reason, the radiological data of the patients were not included in the modeling. This situation can easily be resolved with prospective multi-center studies.

## 5. Conclusions

As a result, it was seen that there studies have been performed using ML methods for AAp and perforated AAp estimation in the literature, but there are no studies combining ML and XAI. Therefore, the present study is the first to combine the ML and XAI models to determine the biochemical blood parameters that predict AAp and perforated AAp. The results will help clinicians identify individuals at risk by paying attention to which biochemical blood parameters in patients with AAp.

## Figures and Tables

**Table 1:** Metrics for classification of AAp.

Metric	Value	95% CI Limits
Accuracy	0.882	0.856–0.908
F1-Score	0.887	0.862–0.913
Sensitivity	0.842	0.798–0.880
Specificity	0.932	0.894–0.959
AUC	0.947	0.913–0.962

**Table 2:** Importance values of factors for classification of AAp.

Feature	Feature Importance (Normalized SHAP Values)
TBil	0.1663400
PNR	0.1600970
PDW	0.1330800
MCV	0.1112450
WBC	0.0924013
CRP	0.0876078
Neutrophil	0.0867753
WNR	0.0599300
WLR	0.0594280
NLR	0.0430910

**Table 3:** Metrics for classification of perforated AAp.

Metric	Value	95% CI Limits
Accuracy	0.92	0.896–0.945
F1-Score	0.911	0.885–0.994
Sensitivity	0.941	0.899–0.969
Specificity	0.905	0.863–0.938
AUC	0.969	0.904–0.987

**Table 4:** Importance values of factors for classification of perforated AAp.

Feature	Feature Importance (Normalized SHAP Values)
CRP	0.265083
PDW	0.112824
Age	0.101890
MPV	0.055570
TBil	0.052502
PLT	0.046732
PLR	0.045915
RDW	0.040585
WBC	0.039910
MCH	0.039698
WLR	0.036890
MCV	0.033890
Lymphocyte	0.030395
NLR	0.030340
Neutrophil	0.026850
PNR	0.022790
WNR	0.018090

## Data Availability

The datasets analyzed during the current study are available from the corresponding author on reasonable request.

## Abbreviations

AAp—Acute appendicitis; NA—Negative appendectomy; AI—Artificial intelligence; XAI—Explainable artificial intelligence; SHAP—Shapley Additive Explanations; SMOTE—Synthetic Minority Over-sampling Technique; ML—Machine learning; AUC—Area under the receiver operator curve; WBC—White blood cell; WLR—White blood cell lymphocyte ratio; WNR—White blood cell neutrophil ratio; NLR—Neutrophil lymphocyte ratio; CRP—C-reactive protein; PLT—Platelete; PNR—Platelet neutrophil ratio; PLR—Platelet lymphocyte ratio; PDW—Platelet distribution width; MPV—Mean platelet volume; TBil—Total bilirubin; RDW- Red blood cell distribution width; MCH—Mean corpuscular hemoglobin; MCV—Mean corpuscular volume; IQR—Interquartile range; CT—Computed tomography; US—Ultrasonography.

## References

[1] Akbulut S., Bahçe Z.S., Öztaş T., Gümüş S., Söğütçü N., Sakarya H., Gök A.F.K., Yağmur Y. (2021). Assessment of demographic, clinical and histopathological features of patients who underwent appendectomy due to a presumed diagnosis of acute appendicitis. Ulus. Travma Acil Cerrahi Derg..

[2] Akbulut S., Koc C., Kocaaslan H., Gonultas F., Samdanci E., Yologlu S., Yilmaz S. (2019). Comparison of clinical and histopathological features of patients who underwent incidental or emergency appendectomy. World J. Gastrointest. Surg..

[3] Koç C., Akbulut S., Coşkun E.I., Sarıcı B., Yılmaz S. (2020). Comparison of the demographic and clinical features of pregnant and non-pregnant patients undergoing appendectomy. Ulus. Travma Acil Cerrahi Derg..

[4] Sarıcı K.B., Akbulut S., Koç C., Tuncer A., Yılmaz S. (2020). Liver transplant versus non-liver transplant patients underwent appendectomy with presumed diagnosis of acute appendicitis: Case-control study. Ulus. Travma Acil Cerrahi Derg..

[5] Lin K.-B., Lai K.R., Yang N.-P., Chan C.-L., Liu Y.-H., Pan R.-H., Huang C.-H. (2015). Epidemiology and socioeconomic features of appendicitis in Taiwan: A 12-year population-based study. World J. Emerg. Surg..

[6] Wickramasinghe D.P., Xavier C., Samarasekera D.N. (2021). The Worldwide Epidemiology of Acute Appendicitis: An Analysis of the Global Health Data Exchange Dataset. World J. Surg..

[7] Akbulut S., Koç C., Şahin T.T., Şahin E., Tuncer A., Demyati K., Şamdancı E., Çolak C., Yılmaz S. (2021). An investigation into the factors predicting acute appendicitis and perforated appendicitis. Ulus. Travma Acil Cerrahi Derg..

[8] Ferris M., Quan S., Kaplan B.S., Molodecky N., Ball C.G., Chernoff G.W., Bhala N., Ghosh S., Dixon E., Ng S. (2017). The Global Incidence of Appendicitis: A Systematic Review of Population-based Studies. Ann. Surg..

[9] Jeon B., Kim H., Heo S. (2019). CT Scan Findings Can Predict the Safety of Delayed Appendectomy for Acute Appendicitis. J. Gastrointest. Surg..

[10] Yang Z., Sun F., Ai S., Wang J., Guan W., Liu S. (2019). Meta-analysis of studies comparing conservative treatment with antibiotics and appendectomy for acute appendicitis in the adult. BMC Surg..

[11] Akmese O., Dogan G., Kor H., Erbay H., Demir E. (2020). The Use of Machine Learning Approaches for the Diagnosis of Acute Appendicitis. Emerg. Med. Int..

[12] Prabhudesai S.G., Gould S., Rekhraj S., Tekkis P.P., Glazer G., Ziprin P. (2008). Artificial neural networks: Useful aid in diagnosing acute appendicitis. World J. Surg..

[13] Unlu C., de Castro S., Tuynman J., Wust A., Steller E., van Wagensveld B. (2009). Evaluating routine diagnostic imaging in acute appendicitis. Int. J. Surg..

[14] Lee Y., Hu P., Cheng T., Huang T., Chuang W. (2013). A preclustering-based ensemble learning technique for acute appendicitis diagnoses. Artif. Intell. Med..

[15] Jeon B.G., Kim H.J., Jung K.H., Lim H.I., Kim S.W., Park J.S., Kim K.H., Kim I.D. (2016). Appendectomy: Should It Be Performed So Quickly?. Am. Surg..

[16] Capoglu R., Gonullu E., Bayhan Z., Coskun M., Harmantepe T., Kucuk F. (2022). Comparison of scoring systems regarding the gender as a parameter with the traditional scoring systems for predicting appendicitis. Updates Surg..

[17] Jose T., Rajesh P.S. (2021). Appendicitis Inflammatory Response Score in Comparison to Alvarado Score in Acute Appendicitis. Surg. J. (N. Y.).

[18] Maghsoudi L.H., Soltanian A., Shirzadi A., Alizadeh-Kashani R., Ahmadinejad M. (2021). Biomarker of urinary 5-HIAA as a valuable predictor of acute appendicitis. Pract. Lab. Med..

[19] Stankovic N., Surbatovic M., Stanojevic I., Simić R., Djuricic S., Milickovic M., Grujic B., Savic D., Marinovic V.M., Stankovic M. (2019). Possible cytokine biomarkers in pediatric acute appendicitis. Ital. J. Pediatr..

[20] Došilović F.K., Brčić M., Hlupić N. (2018). Explainable artificial intelligence: A survey. Proceedings of the 2018 41st International Convention on Information and Communication Technology, Electronics and Microelectronics (MIPRO).

[21] Sundararajan M., Najmi A. (2020). The Many Shapley Values for Model Explanation. Proceedings of the 37th International Conference on Machine Learning.

[22] Ozen H., Bal C. (2020). A study on missing data problem in random Forest. Osman. Tıp Derg..

[23] Chawla N.V., Bowyer K.W., Hall L.O., Kegelmeyer W.P. (2002). SMOTE: Synthetic minority over-sampling technique. J. Artif. Intell. Res..

[24] He H., Garcia E.A. (2009). Learning from imbalanced data. IEEE Trans. Knowl. Data Eng..

[25] Ahmadpour H., Bazrafshan O., Rafiei-Sardooi E., Zamani H., Panagopoulos T. (2021). Gully Erosion Susceptibility Assessment in the Kondoran Watershed Using Machine Learning Algorithms and the Boruta Feature Selection. Sustainability.

[26] Prokhorenkova L., Gusev G., Vorobev A., Dorogush A.V., Gulin A. (2018). CatBoost: Unbiased boosting with categorical features. Proceedings of the 32nd International Conference on Neural Information Processing Systems 2018.

[27] Dorogush A.V., Ershov V., Gulin A. (2018). CatBoost: Gradient boosting with categorical features support. arXiv.

[28] Jabeur S.B., Gharib C., Mefteh-Wali S., Arfi W.B. (2021). CatBoost model and artificial intelligence techniques for corporate failure prediction. Technol. Forecast. Soc. Chang..

[29] Bakhareva N., Shukhman A., Matveev A., Polezhaev P., Ushakov Y., Legashev L. (2019). Attack detection in enterprise networks by machine learning methods. Proceedings of the 2019 international Russian Automation Conference (RusAutoCon).

[30] Gunning D., Stefik M., Choi J., Miller T., Stumpf S., Yang G.Z. (2019). XAI-Explainable artificial intelligence. Sci. Robot..

[31] Samek W., Müller K.-R. (2019). Towards explainable artificial intelligence. Explainable AI: Interpreting, Explaining and Visualizing Deep Learning.

[32] Tjoa E., Guan C. (2021). A Survey on Explainable Artificial Intelligence (XAI): Toward Medical XAI. IEEE Trans. Neural Netw. Learn. Syst..

[33] Rodríguez-Pérez R., Bajorath J. (2020). Interpretation of machine learning models using shapley values: Application to compound potency and multi-target activity predictions. J. Comput. Aided Mol. Des..

[34] Lundberg S., Lee S.-I. A unified approach to interpreting model predictions. Proceedings of the 31st International Conference on Neural Information Processing Systems 2017.

[35] Vandenbroucke J.P., von Elm E., Altman D.G., Gøtzsche P.C., Mulrow C.D., Pocock S.J., Poole C., Schlesselman J.J., Egger M., STROBE Initiative (2014). Strengthening the Reporting of Observational Studies in Epidemiology (STROBE): Explanation and elaboration. Int. J. Surg..

[36] Busnatu Ș., Niculescu A.-G., Bolocan A., Petrescu G.E., Păduraru D.N., Năstasă I., Lupușoru M., Geantă M., Andronic O., Grumezescu A.M. (2022). Clinical Applications of Artificial Intelligence—An Updated Overview. J. Clin. Med..

[37] Nakamura T., Sasano T. (2022). Artificial intelligence and cardiology: Current status and perspective. J. Cardiol..

[38] Xia J., Wang Z., Yang D., Li R., Liang G., Chen H., Heidari A.A., Turabieh H., Mafarja M., Pan Z. (2022). Performance optimization of support vector machine with oppositional grasshopper optimization for acute appendicitis diagnosis. Comput. Biol. Med..

[39] Mijwil M.M., Aggarwal K. (2022). A diagnostic testing for people with appendicitis using machine learning techniques. Multimed. Tools Appl..

[40] Reismann J., Romualdi A., Kiss N., Minderjahn M.I., Kallarackal J., Schad M., Reismann M. (2019). Diagnosis and classification of pediatric acute appendicitis by artificial intelligence methods: An investigator-independent approach. PLoS ONE.

[41] El Hechi M.W., Eddine S.A.N., Maurer L.R., Kaafarani H.M.J.S. (2021). Leveraging interpretable machine learning algorithms to predict postoperative patient outcomes on mobile devices. Surgery.

